# Effect of Media Usage Selection on Social Mobilization Speed: Facebook vs E-Mail

**DOI:** 10.1371/journal.pone.0134811

**Published:** 2015-09-30

**Authors:** Jing Wang, Stuart Madnick, Xitong Li, Jeff Alstott, Chander Velu

**Affiliations:** 1 Management Science and Engineering, Harbin Institute of Technology, Harbin, China; 2 Sloan School of Management and School of Engineering, Massachusetts Institute of Technology, Cambridge, Massachusetts, United States of America; 3 Department MOSI, HEC Paris, Jouy-en-Josas, France; 4 Brain Mapping Unit, University of Cambridge, Cambridge, United Kingdom; 5 Institute for Manufacturing, Department of Engineering, University of Cambridge, Cambridge, United Kingdom; University of Warwick, UNITED KINGDOM

## Abstract

Social mobilization is a process that enlists a large number of people to achieve a goal within a limited time, especially through the use of social media. There is increasing interest in understanding the factors that affect the speed of social mobilization. Based on the Langley Knights competition data set, we analyzed the differences in mobilization speed between users of Facebook and e-mail. We include other factors that may influence mobilization speed (gender, age, timing, and homophily of information source) in our model as control variables in order to isolate the effect of such factors. We show that, in this experiment, although more people used e-mail to recruit, the mobilization speed of Facebook users was faster than that of those that used e-mail. We were also able to measure and show that the mobilization speed for Facebook users was on average seven times faster compared to e-mail before controlling for other factors. After controlling for other factors, we show that Facebook users were 1.84 times more likely to register compared to e-mail users in the next period if they have not done so at any point in time. This finding could provide useful insights for future social mobilization efforts.

## Introduction

Online media has the ability to mobilize a large number of people to achieve a goal in limited time [1]. The process that enlists people to complete tasks has been called social mobilization. Social mobilization with the Internet has been used to map crisis event in real time [2] and operate search-and-rescue actions [3]. It is also an important way to participate in political decision-making [4, 5]. In many of these social mobilization tasks, speed is a top concern [6]. Because of its increasing importance, research has been done to understand the process both theoretically and practically [6]. Recently, researchers have analyzed some of the factors that influence mobilization speed [7]. However, few have investigated the effect of different communication channels on social mobilization speed (see [6, 8, 9]). Despite the lack of empirical analysis on this topic, many have assumed that the use of Facebook would lead to faster mobilization [10, 11]. In this paper, we explore the difference of social mobilization speed between those who chose to use Facebook and those who used e-mail. In this paper, we address the following research questions:

- Is there a difference in mobilization speed between Facebook and e-mail users?
- If so, what is the magnitude of the difference in mobilization speed between Facebook and e-mail users?

Social mobilization speed is influenced by various factors. Timing is one of them. For different social media, the daily number of new posts for weekdays and weekends shows different patterns [12]. Participants also modulate their activities following a daily cycle (daytime vs. night) [13]. Meanwhile, the closer to the contest start date, the faster the mobilization speed [7]. Gender has been shown to have an impact on information diffusion; for example, men are more likely than women to receive a given message [14] which in turn could influence social mobilization. In addition, the speed by which information spreads varies with the age of participants [15]. Furthermore, information source has significant homophily influence on social mobilization [7]. Thus, these factors might explain the difference in mobilization speed between users of different communication channels such as Facebook and e-mail respectively. In this paper, we examine the difference in social mobilization speed between users of Facebook and e-mail, after controlling for other influencing factors (gender, age, timing, and the homophily of information source). We find that the mobilization speed attained by users of Facebook remains significantly faster than that of e-mail both before and after controlling for other influencing factors. We believe that these findings are useful for improving performance of social mobilization tasks.

## Results

### Data

For our social mobilization analyzes, we used the Langley Knights competition data set [7]. The competition involved locating 5 knights in shining armor—three real knights and two virtual ones. The three ‘real’ knights wore armor and stood in their appointed venue in unspecified public parks in England from 9am to 9pm each day. The two ‘virtual’ ones were to be found on Google Maps or Google Earth. This worldwide competition started on July 2, 2011, but the recruiting of members for teams started about a month beforehand. Participants who registered could invite their friends to join the contest as new team members through any way that they wished, though facilities were provided to make e-mail forwarding and Facebook messaging easy—over 80% of the participants who reported how they were recruited used these methods. In particular, participants who registered using Facebook Connect could, at the end of the registration process, invite their Facebook friends to join the contest under their team. Registered participants were also provided with a URL and an e-mail acknowledgement that they could share with others, such as via e-mail forwarding, to register through, which would automatically put those new participants on their recruiter’s team.

We note that the competition was conceived and conducted by Langley Castle and was not conducted for research purposes and was not subject to IRB review. The data was provided to the authors by Langley Castle in anonymized manner after the conclusion of the competition. The authors did not gather the data; and no names were provided as part of the data, so the data was already anonymized.

A total of 1,077 participants registered, with 148 starting their own team. The registration system was kept open for a short duration after the contest ended to ensure all team registration information was in order. As a result of this, however, there were 12 participants that registered after the contest ended. Therefore, although there were 1089 participants, we dropped the 12 participants who registered after the contest ended and used the data from 1077 participants.

Of the 148 teams, 97 did not mobilize any other team members, leaving 51 teams that recruited new participants. Participants could act as both recruits (if they joined a team) and recruiters (if they mobilized others). In these teams, 152 participants acted as recruiters, mobilizing at least one other participant. These recruiters mobilized 929 recruits (see S1 Data). The mean team size was 7.05, and the mean size of teams larger than 1 was 18.55.

For each participant, the date and time of his/her registration was recorded by the system. Demographic information (age, gender, etc.) and which communication channel was used to contact them were collected during the registration process. Because participants were not required to answer any question, there was some missing information in the dataset. Of those reporting the method by which they were recruited, 48.6% of participants were recruited through e-mail and 35.5% were recruited through Facebook. Those two media accounted for around 84.1% of all the participants reporting their method of recruitment.

### Mobilization speed difference between Facebook and e-mail

**Fig 1:**
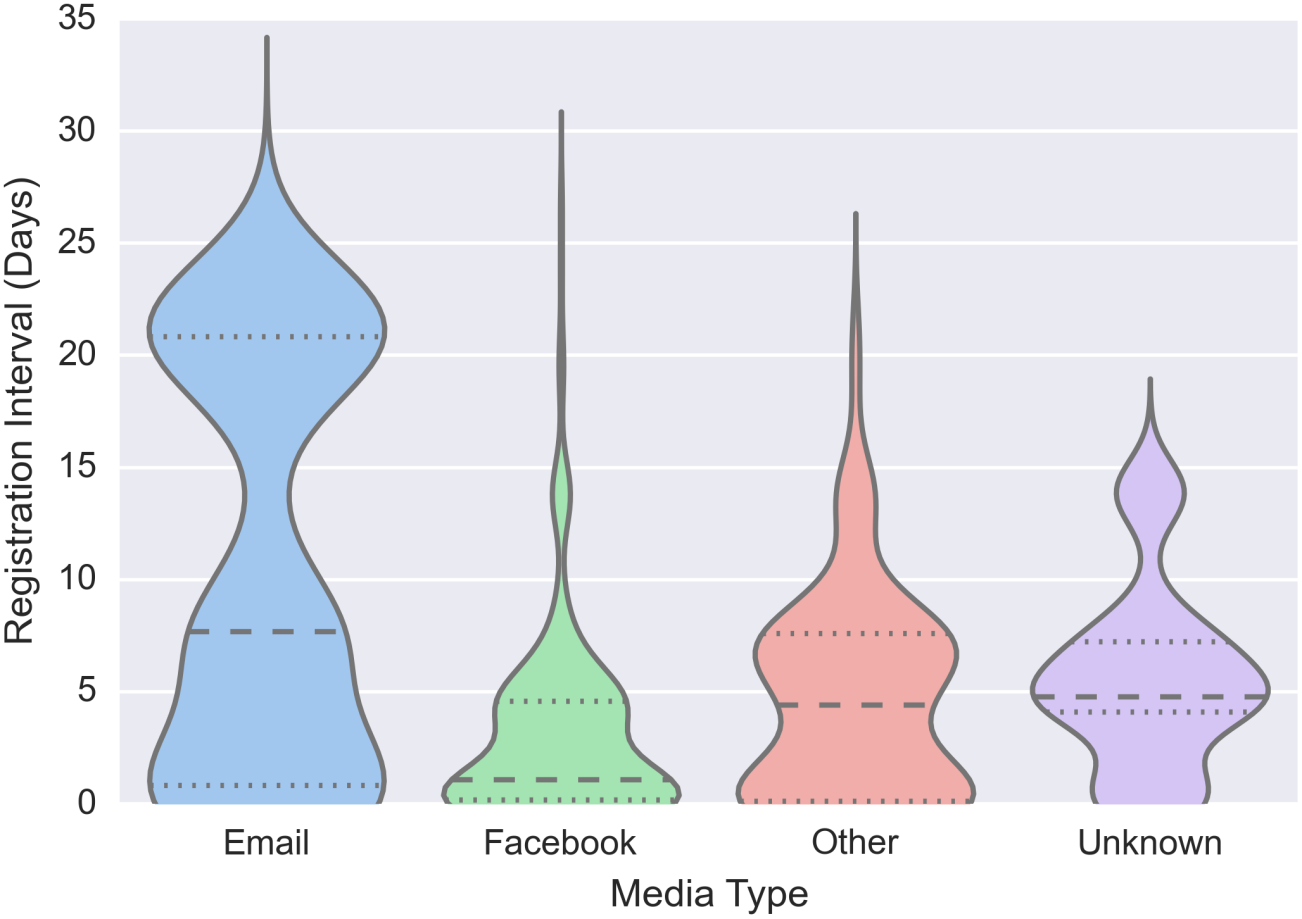
The mobilization speed of Facebook users was faster than that of e-mail users. Other social media communications methods reported included: Instant messaging, phone call, text message, other social media (e.g., Twitter), and word of mouth. Indirect methods reported included Langley Knights competition web site, newspaper, television, and radio.

Following Alstott et al. [7], the mobilization speed was defined as the interval days from the registration time of one participant to that of their recruit. The mobilization speeds of four categories of recruitees are shown in Fig 1: Those that were recruited using (i) e-mail, (ii) Facebook, (iii) some other media (e.g., telephone, word-of-mouth), and (iv) those that did not report the method by which they were recruited.

As can be seen, not only are people contacted through Facebook recruited fasted, they are recruited substantially faster, with a median mobilization speed of 1.08 days for Facebook compared with 7.68 days for e-mail, making mobilization through using Facebook more than seven times faster compared to e-mail.

In our further analysis below, we only used those participants that were recruited through Facebook/e-mail and provided complete information during the registration process. Out of the 929 recruits, 597 used either Facebook or e-mail. The remainder used other social media communication methods or did not report the media they used. The number of participants that used Facebook or e-mail who provided completed information was 322. Some characteristics and differences between the users of these media include:

- Among e-mail users, the percentage of recruits who are female (32.5%) is much smaller than that among Facebook users (62.9%).
- The percentage of recruits who were contacted through the same source as their recruiters in the e-mail group (30.7%) is smaller than that in the Facebook group (47.8%).
- The distribution of age of e-mail users is similar to that of Facebook users.

The finding that users of Facebook were mobilized faster than users of e-mail remained even after controlling for the above factors, as shown through the empirical analysis in the next section.

We conducted normality tests and find that both of the significant values of the Shapiro-Wilk Test are below 0.05, which indicates that the data significantly deviate from a normal distribution. Since the mobilization speed values does not show a normal distribution, we used a non-parametrical test, Mann-Whitney U-test, to check the difference between Facebook and e-mail in mobilization speed, where we compare the mean ranks of the mobilization speed. All the observations are ranked from the lowest to the highest for each group (Facebook and e-mail), and then the sum of those ranks are calculated for each group. The mean rank is the mean of the total rank [16]. The results show that mobilization speed in the Facebook group was significantly faster statistically than that in the e-mail group (mean rank, 180.53 vs. 141.99; z = -3.714, P < 0.001).

**Fig 2:**
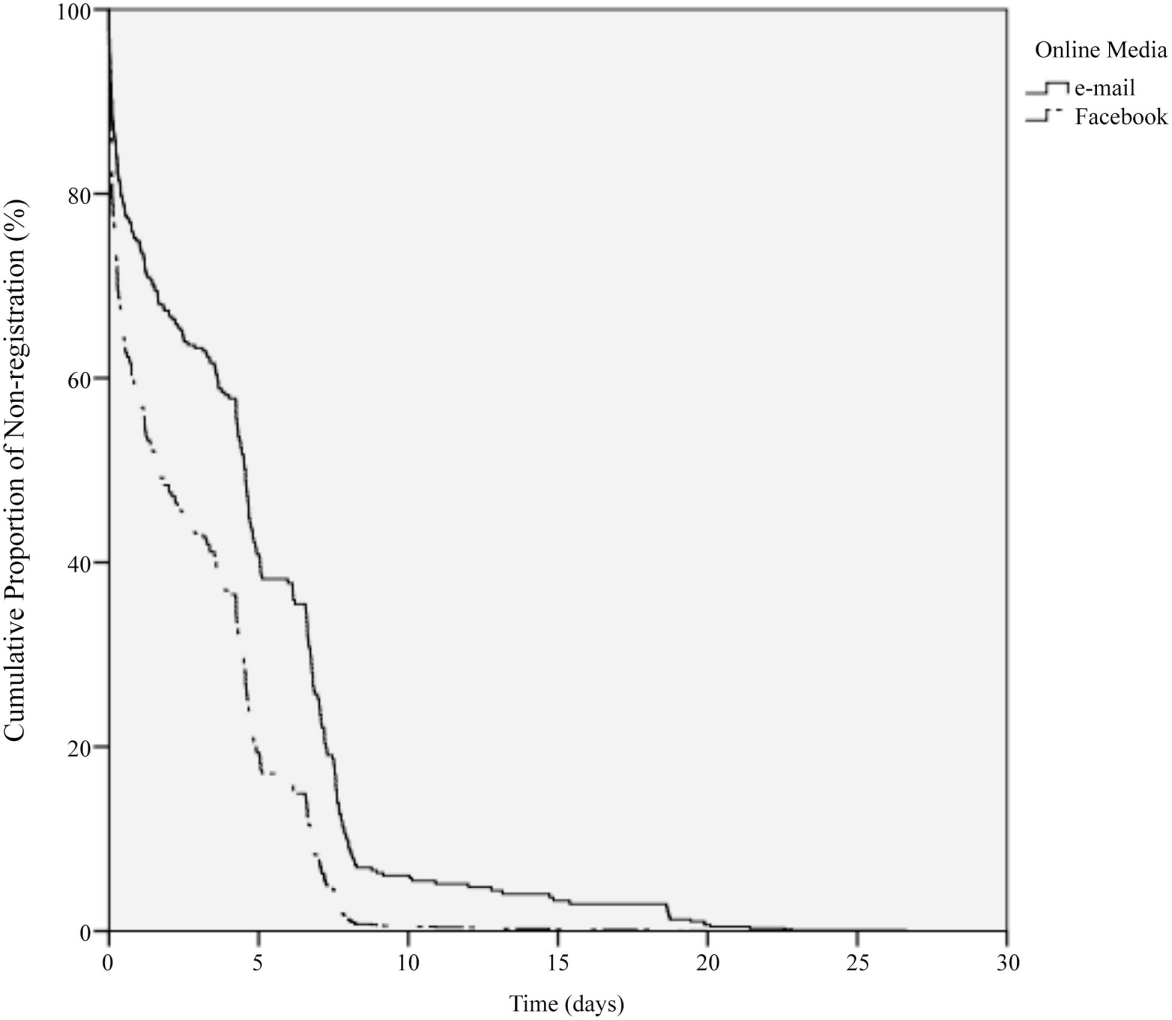
The cumulative proportion of non-registration among Facebook users decreases faster than that among e-mail users, which indicates that users of Facebook were mobilized faster than users of e-mail. The dashed curve estimates the cumulative proportion of Facebook users (the solid curve estimates e-mail users) who have not registered for the competition.

The corresponding Kaplan-Meier type survival curve which plots the cumulative proportion of non-registration among Facebook and e-mail users respectively over time is shown in Fig 2. Among e-mail users, the cumulative proportion of people who have not registered for the competition decreases slower than that among Facebook users. Therefore, the analysis shown in Fig 2 also supports our key result that users of Facebook were mobilized faster than users of e-mail. However, as noted above, there are other traits influencing the mobilization speed, such as gender, age, etc. The goal of our study was to understand the mobilization speed difference between Facebook and e-mail, after controlling for other confounding factors which we turn to in the next section.

### Analysis of mobilization speed using Cox proportional hazard model

We analyze the influence of social media on mobilization speed with a Cox proportional hazard model (see Methods in S1 File), which is the standard method for the evaluation for social contagion in sociology [17]. We also include several control variables in the regression in order to isolate the effect of these variables on mobilization speed. These control variables include timing [12, 13], gender [14], and age [15]. Timing factors include workweek/weekend [12], daytime/night [13], and time left till the end of the contest [7]. Gender factor consists of male and female. Age factor is divided into four different age groups: youth (20 years old and under), young adults (21–40 years old), middle-aged adults (41–60 years old), and seniors (over 60 years old). Considering that information source has significant homophily influence [7], we included another control variable: if the recruit heard through the same medium as the recruiter (e.g., both were contacted via e-mail). Below we first discuss the effects of online media (Facebook compared to e-mail), and then add the above control variables step by step. These independent variable codes are described in Table 1. For Cox proportional hazard analysis, we used the data from the 322 who used either Facebook or e-mail and provided complete information.

**Table 1:** Independent variable codes.

Independent variables	Dummy variables	Definition and codes
Online media		1(Facebook), 0(E-mail)
Media homophily		1(yes: the recruit heard through same medium as his/her recruiter),0(no: the recruit heard through different medium from his/her recruiter)
Workweek		1(workweek), 0(weekend)
Daytime		1(daytime), 0(night)
TimeLeft		The number of days until the competition ended.
Gender		1(female), 0(male)
Age	Age_Young	1(young adults: 21–40), 0(otherwise)
	Age_Middle	1(middle-aged adults: 41–60), 0(otherwise)
	Age_Senior	1(seniors: >60), 0(otherwise)

A hazard ratio (HR) is the ratio of the probability of an outcome event in the exposed group compared with that in the non-exposed group in a period of time [18]. In our analysis, the hazard ratio of non-exposed group (e-mail group) equals one. The exposed group (Facebook group) is faster at registering for the contest than the non-exposed group, if its hazard ratio is larger than one. Conversely, a hazard ratio that is smaller than one reflects slower mobilization speed. Table 2 shows the results of the effects of social media on mobilization speed. The choice of social media has a significant impact on mobilization speed (Sig. < 0.05) whereby; the social mobilization speed of Facebook is faster than that of e-mail (HR > 1).

**Table 2:** Online media factor has significant influence on mobilization speed.

	B	S.E.	Wald	df	Sig.	HR	95.0% CI for HR	
							Lower	Upper
Online media	0.645	0.120	28.951	1	0.000	1.906	1.506	2.411

The results include the coefficient of the estimated regression equation (B), their standard errors (S.E.), Wald statistics (Wald), the degree of freedom (df), significance (Sig.), hazard ratio (HR), and confidence intervals (CI) for the 95% significance level (95.0% CI for HR). The hazard ratio of online media (1.906) is larger than one, meaning that the social mobilization speed of Facebook is faster than that of e-mail.

### Results after isolating the effect of control variables

**Fig 3:**
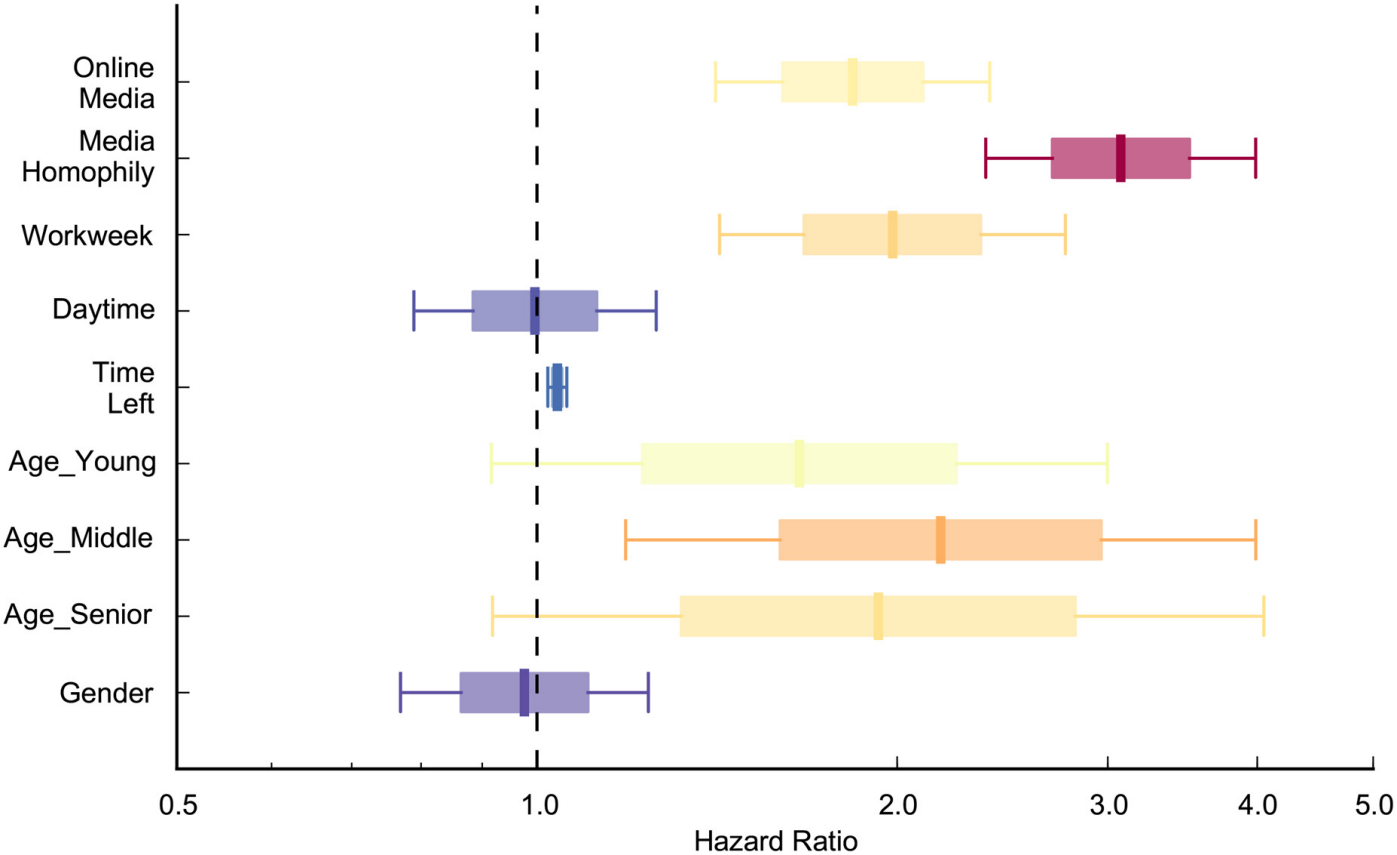
The social mobilization speed of Facebook users is faster significantly than that of e-mail users in control of media homophily, workweek, daytime, time left, age, and gender factors. Boxes correspond to standard errors and whiskers are 95% confidence intervals. The redder box is, the faster mobilization speed (higher hazard ratio) is. On the other hand, slower mobilization (lower hazard ratio) was represented by bluer boxes. The hazard ratio of online media is 1.836, which is larger than one. It indicates that Facebook recruit people faster than e-mail (p<0.01). Mobilization was faster when recruit and his/her recruiter heard through the same media compared to when they heard through different media (p<0.01). People were more likely to register when it is a workday than it is a weekend (p<0.01). There are no significant differences in mobilization speed between daytime and night, or between female participants and male participants. The mobilization speed is slower when it is closer to the contest end date (p<0.01). Compared to youth, young adults, middle-aged adults, and seniors were recruited faster (p<0.1).

As discussed above, some studies have shown that same information source [7], timing (workweek/weekend [12], daytime/night [13], and time left [7]), age [15], and gender [14] influence mobilization speed. To isolate the effect of these control variables, we include them step by step as shown in Models 1–6 in Table 3. It indicates that the mobilization speed of Facebook is faster than that of e-mail, even after controlling for these control variables in the model (all the HR values of ‘online media’ are larger than one). Facebook users were 1.84 times more likely to register compared to e-mail users to register in the next period if they have not done so at any point in time (HR = 1.836) as shown in Model 6 which includes all the controls variables (for goodness of fit measures, see S2 File). The final results (for Model 6) are shown diagrammatically in Fig 3.

**Table 3:** Online media has significant influence on mobilization speed in control of media homophily, workweek, daytime, time left, age, and gender factors.

	Model 1	Model 2	Model 3	Model 4	Model 5	Model 6
Variables	HR	HR	HR	HR	HR	HR
	(B, S.E.)	(B, S.E.)	(B, S.E.)	(B, S.E.)	(B, S.E.)	(B, S.E.)
**Online media**	**1.746** ***	**1.684** ***	**1.700** ***	**1.738** ***	**1.823** ***	**1.836** ***
	**(0.557, 0.122)**	**(0.521, 0.124)**	**(0.531, 0.125)**	**(0.552,0.126)**	**(0.600,0.131)**	**(0.608, 0.135)**
Media homophily	2.351***	2.814***	2.802***	2.985***	3.089***	3.076***
	(0.855, 0.122)	(1.035, 0.129)	(1.030, 0.129)	(1.094, 0.130)	(1.128, 0.131)	(1.124, 0.132)
Workweek		1.959***	1.954***	2.109***	1.982***	1.983***
		(0.672, 0.164)	(0.671, 0.164)	(0.746, 0.167)	(0.684, 0.170)	(0.684, 0.170)
Daytime			1.058	1.033	1.000	0.996
			(0.056, 0.114)	(0.032, 0.115)	(0.000, 0.118)	(-0.004, 0.119)
Time left				1.038***	1.040***	1.040***
				(0.038, 0.009)	(0.039, 0.009)	(0.039, 0.009)
Age_Young					1.661*	1.657*
					(0.508, 0.302)	(0.505, 0.302)
Age_Middle					2.177**	2.175**
					(0.778, 0.309)	(0.777, 0.309)
Age_Senior					1.936*	1.929*
					(0.661, 0.378)	(0.657, 0.379)
Gender						0.976
						(-0.024, 0.122)
Number of observations	322	322	322	322	322	322

Dependent variable is mobilization speed. In each step, HR of online media is larger than one. It indicates that Facebook has faster mobilization speed compared to e-mail.

*p < 0.10,

**p < 0.05,

***p < 0.01.

We also find that mobilization speed was faster when there was media homophily, that is that the recruits heard through same medium as their recruiters, compared to when media homophily was not present (HR = 3.076 > 1).

### Robustness analysis for missing values

**Fig 4:**
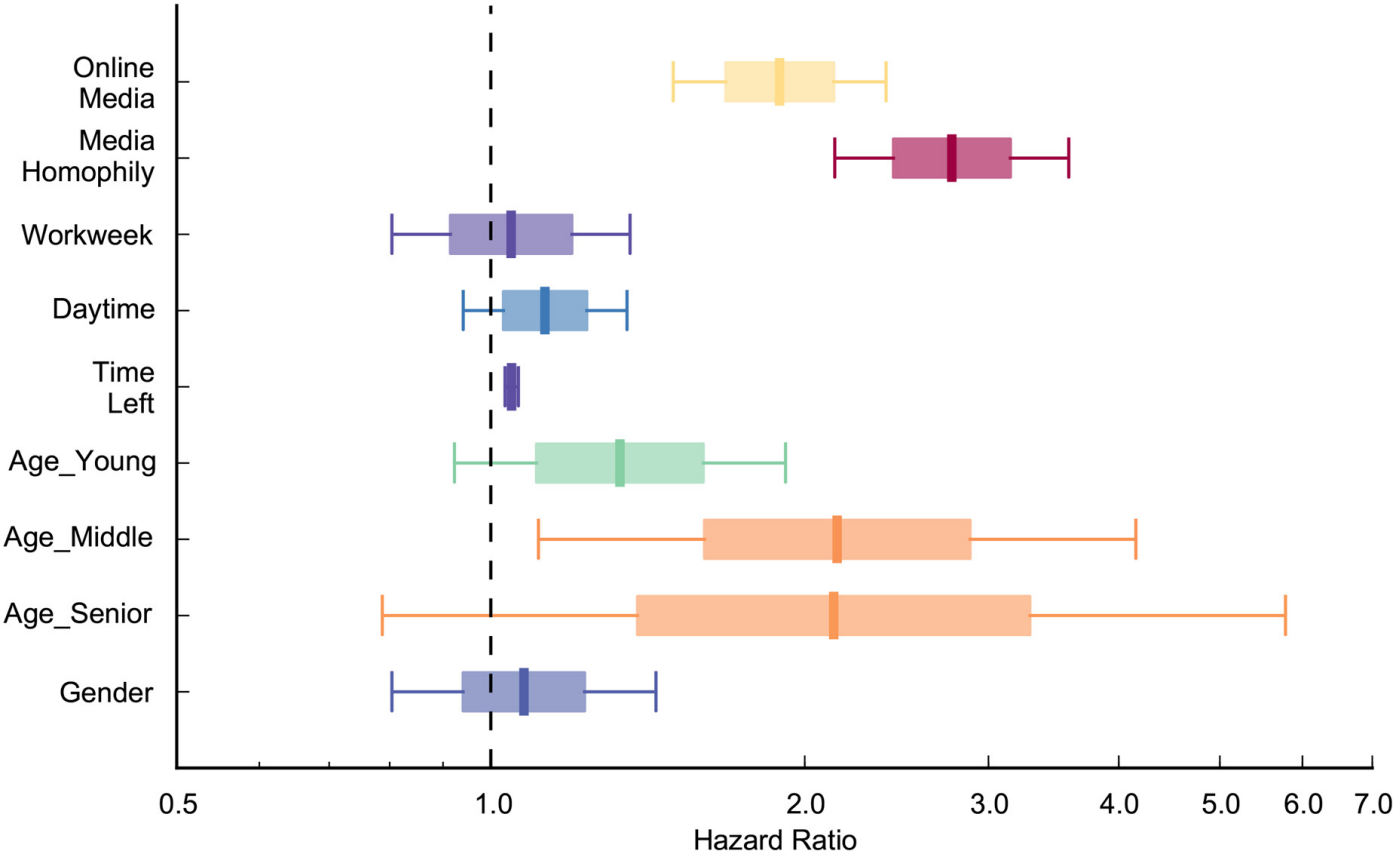
The result that Facebook recruits people faster than e-mail is robust after imputing values of missing data. The hazard ratio of online media is significantly larger than one controlling for media homophily, workweek, daytime, time left, age, and gender factors (HR = 1.892, p<0.01). It indicates that the mobilization speed of Facebook users is faster than that of e-mail users. Mobilization speed was much faster when media homophily is present, compared to when there is no media homophily (p<0.01). There are no significant differences in mobilization speed between workday and weekend, daytime and night, or between women and men. The closer to the contest end date, the slower the mobilization speed (p<0.01). Middle-aged adults were recruited faster than youth (p<0.05).

**Fig 5:**
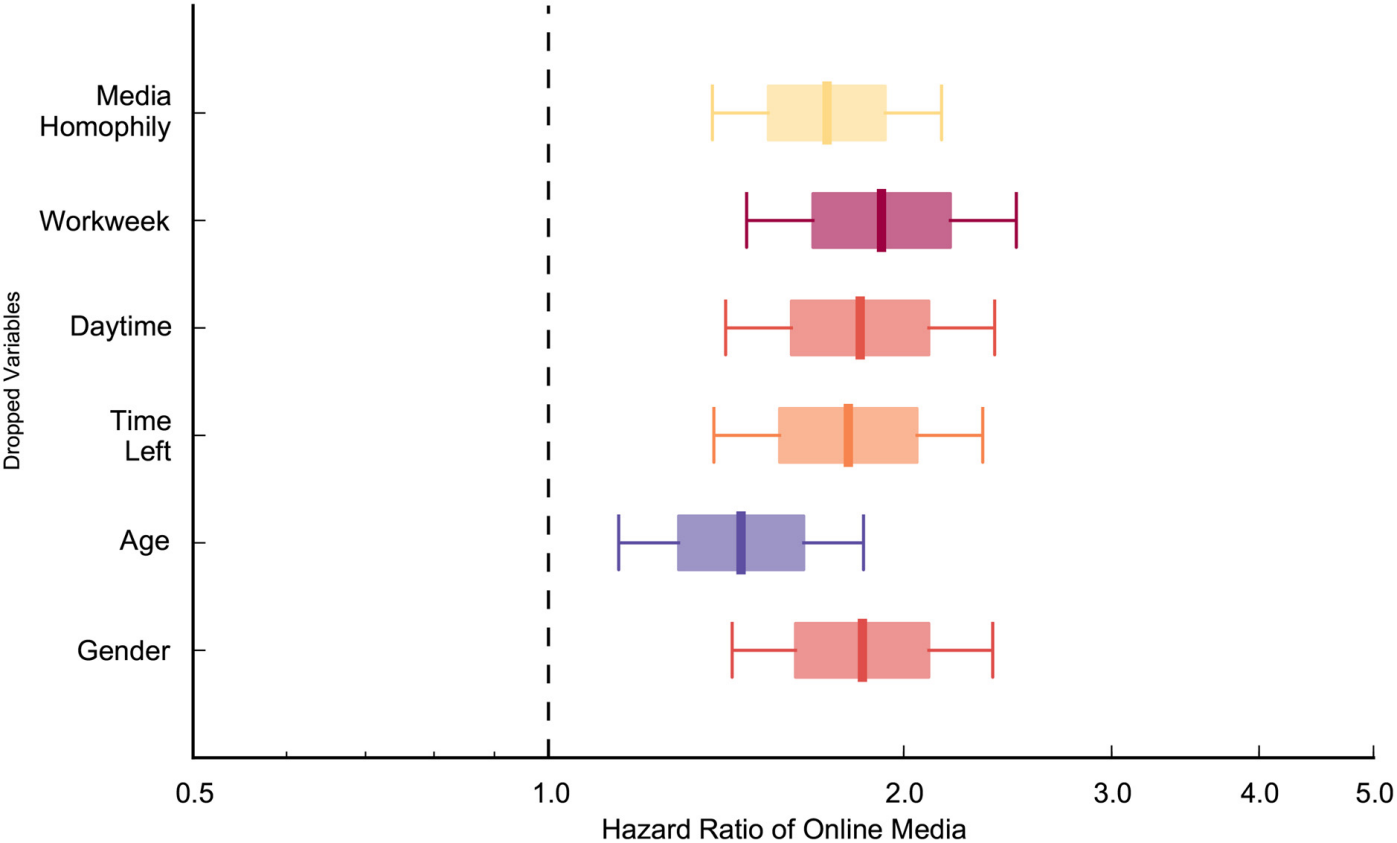
The result that Facebook recruits people faster than e-mail is robust after dropping one variable at a time. All the hazard ratios of online media are larger than one when dropping one control variable at a time and controlling for the remainder control variables (p<0.01). It indicates that the mobilization speed of Facebook users is faster than that of e-mail users consistently.

For the analysis shown in Models 1–6 of Table 3, we used the data for 322 participants that provided full information. However, there were 275 participants who used either Facebook or e-mail that did not provide information on one or more of the control variables we used in the Cox proportional hazard analysis. To address these missing values, we conducted two further analyses to test for the robustness of our results. The first uses an imputation method for missing values using logistic regression to impute the values of missing data and then run the Cox proportional hazard model on the full-imputed dataset. The result that the mobilization speed of Facebook users was faster than that of e-mail users is robust under this method of imputation of missing values. In particular, the hazard ratio of online media is larger than one and almost the same with the analysis using the complete data as shown in Fig 4. In the second approach, we drop one variable at a time from Model 6 of Table 3 in order to increase the number of observations/participants and hence account for missing data. The results are also robust with that using only the complete dataset as Facebook users are faster than e-mail uses as shown in Fig 5.

## Discussion

Social mobilization is increasing in importance as social media penetrates into every aspect of social life. The speed of mobilization has attracted a lot of attention because of its broad social influence. Also, mobilization speed is often critical to the final outcome; for instance, time is of the essence in a rescue movement. We examined differences in mobilization speed between users of e-mail and Facebook in order to better understand the factors that influence the speed of mobilization. We found that Facebook mobilized people significantly faster than e-mail after controlling for other factors that may also influence the mobilization speed.

There are many plausible explanations for our finding. Facebook is more public compared to e-mail as the latter is usually used for private communications [19]. For example, Facebook posts status updates from members immediately to their respective friends and also enable such friends to share opinions through their comments. Therefore, arguably positive opinion about an event (such as the competition) could enhance and speed up the feedback in Facebook faster than in e-mail [20]. Furthermore, people usually login to their Facebook account very frequently compared to only 70% of users that check their e-mail daily which raises the possibility of people obtaining information about the competition sooner on Facebook compared to e-mail [21]. Hence, the characteristics of Facebook (public, feedback attribute, and high checking frequency) may contribute to the findings that social mobilization through Facebook is faster than that through e-mail.

Studies have shown that most Facebook users know each other in real-life first and then become friends on Facebook [22]. In contrast, for e-mail, many relationships between users are constructed in the opposite direction whereby the majority of e-mail users build connections through the Internet first and then migrate to online e-mail relationships. Moreover, people commonly use Facebook for non-professional and informal purposes and e-mail for professional and formal reasons [23]. Thus, Facebook seems to build a more trustful and personal network compared to e-mail, which might lead to faster social mobilization.

The social presence model argues that the features and attributes of the media could also influence the speed of mobilization and posits that the media chosen might vary according to the degree of presence of other individuals [24]. The media with a high degree of presence of other individuals will be more actively used over media with lower degree of presence. In this context, Facebook is typically seen as having higher social presence compared to e-mail and hence, might display faster mobilization. Similarly, media richness theory posits that communications medium can be from ‘lean’ to ‘rich’ based on the technologies intrinsic properties [25]. Richer media are better at communicating complex and ambiguous information whilst lean media is better at communicating simpler and less ambiguous information. Using media richness theory, one can argue that Facebook is a richer media and might be a better medium to communicate complex propositions/ambiguous message (e.g., arguably invitation to join the Langley Knights competition can be considered to be fairly complex) compared to e-mail which is a relatively less rich media and hence, more suitable to communicate less complex/ambiguous messages. Hence, the response to Facebook is faster than e-mail.

In conclusion, quantitative studies of social mobilization speed are rare and, to the best of our knowledge, the existing studies about social mobilization have not measured the effects of different communication media used (see [6, 8, 9]). Although many people may have assumed that usage of Facebook would lead to faster mobilization, this study provides empirical evidence as well as a measure of the magnitude of the difference. By measuring such factors that predict social mobilization speed, this work advances our understanding of the important phenomenon of the speed of social mobilization.

## Supporting Information

S1 Data Anonymized dataset used to produce the reported analyses. (XLS)

S1 File Methods. (DOCX)

S2 File Goodness of fit measures for the Cox proportional hazards model. (DOCX)
