# Supplementary material for: Effect of Media Usage Selection on Social Mobilization Speed: Facebook vs E-Mail
Source: PLoS One. 2015 Sep 30;10(9):e0134811. doi: 10.1371/journal.pone.0134811 (PMC4589319; doi:10.1371/journal.pone.0134811)
Supplement: S1 File — (DOCX) [file pone.0134811.s002.docx]

**Methods**

Cox proportional hazard model (1972) is the popular method for analyzing information diffusion in marketing and sociology [[1](#_ENREF_1)]. It is also the most widely used method of survival analysis which typically explores the relationship of the survival distribution to covariates [[2](#_ENREF_2)]. For example, in our cases, ‘death’ refers to the registration for the contest and covariates are the influencing factors of mobilization speed. The hazard function (*h*(*t*)) is a key concept of cox model. It is the probability that an object will be dead within a time interval given that the object has been alive up to the beginning of the interval [[3](#_ENREF_3)]. It could seem as the risk of dying at time *t*. In our model, the hazard function is the possibility of registering at time *t*. It can be evaluated using formula:

$$h(t)=\frac{f(t)}{S(t)}$$

Here, *f*(*t*) is the number of objects who are dead in interval beginning at *t*, and *S*(*t*) is the product of the number of objects who are alive at time *t* and the interval width. Cox proportional hazard regression model could be described as the following formula [[2](#_ENREF_2)].

In the formula, *i* is a subscript for object, and the *x*’s are the covariates. The constant *α* is the log-baseline hazard ($\alpha=\log(h_{0}(t))$) . *β* represents the hazard ratios (HR), which is the ratio of the hazard rates corresponding to the conditions described by two levels of an independent variable. It could be presented simply as follows. *h*_A_(*t*) is the risk of registering in the group where independent variable equals A and *h*_B_(*t*) is the risk of registering in group B.

$$HR=\frac{h_{A}(t)}{h_{B}(t)}$$

# References

1. Aral S, Walker D. Identifying influential and susceptible members of social networks. Science. 2012;337(6092):337-41. doi: 10.1126/science.1215842.

2. Fox J. Cox proportional-hazards regression for survival data. Appendix to An R and S-PLUS Companion to Applied Regression. 2002. Available: https://socserv.socsci.mcmaster.ca/jfox/Books/Companion-1E/appendix-cox-regression.pdf. Accessed 6 June 2014.

3. Walters SJ. What is a Cox model? Hayward Medical Communications; 2009. Available: http://www.whatisseries.co.uk. Accessed 6 June 2014.
