# Supplementary material for: Effect of Media Usage Selection on Social Mobilization Speed: Facebook vs E-Mail
Source: PLoS One. 2015 Sep 30;10(9):e0134811. doi: 10.1371/journal.pone.0134811 (PMC4589319; doi:10.1371/journal.pone.0134811)
Supplement: S2 File — (DOCX) [file pone.0134811.s003.docx]

**Goodness of fit**

When we involve all the control variables in the model (Model 6), the model fits the data well with an R-Square of 0.304. We performed several goodness-of-fit tests for the Cox proportional hazards model. The model has nine degrees of freedom. The results of all the tests show that the model fits the data better than a null model. We also calculated the concordance probability, which is used to assessing the discriminatory power and the predictive accuracy of Cox proportional hazards models [[1](#_ENREF_1)], the model has a good probability with 0.698.

| Metric | Value |
| --- | --- |
| Likelihood ratio test | 116.8 (p<0.0001) |
| Wald test | 115 (p<0.0001) |
| Score (logrank) test | 121.9 (p<0.0001) |

**Proportional hazards assumption testing**

The Cox proportional hazards model holds an assumption that the covariates in the hazard function do not vary with time. The scaled Schoenfeld residuals for all explanatory variables are plotted to test this assumption. The proportional hazards assumption has been supported by the lack of linear trends for any of these residuals.

|  |  |  |
| --- | --- | --- |
|  |  |  |
|  |  |  |

# References

1. Gönen M, Heller G. Concordance probability and discriminatory power in proportional hazards regression. Biometrika. 2005;92(4):965-70.
